# Incomplete Stevens-Johnson Syndrome Caused by Sulfonamide Antimicrobial Exposure

**DOI:** 10.5811/westjem.2019.4.42551

**Published:** 2019-05-29

**Authors:** Nikki B. Canter, Lane M. Smith

**Affiliations:** Wake Forest School of Medicine, Department of Emergency Medicine, Winston-Salem, North Carolina

## Abstract

Stevens-Johnson syndrome (SJS) is a mucocutaneous reaction typically brought on by medications or infections. The diagnosis of SJS is typically made when patients present with a variable appearing rash and involvement of the oral, ocular, or genital mucosa. However, there are rare reports of atypical or incomplete SJS. These cases are usually associated with children infected with *Mycoplasma pneumoniae*, which presents with severe mucositis but no rash. Herein, we report the first case of adult incomplete SJS brought on by sulfonamide antimicrobial use without clinical or laboratory evidence of *M. pneumoniae* infection.

## INTRODUCTION

Stevens-Johnson syndrome (SJS) is a rare, mucocutaneous reaction that affects two to seven per million people per year and is often precipitated by medications and infections.^1^ Women, human immunodeficiency virus-infected patients, and those with cancer are disproportionately affected.^2^,^3^ It is characterized by skin rash and the involvement of the oral mucosa, genitals, or conjunctivae, but rare presentations of incomplete SJS are reported in children after *Mycoplasma pneumoniae* infection.^4^ Here, we report the first case of oral and ocular SJS without skin lesions in a healthy adult after exposure to an antibacterial sulfonamide without *M. pneumoniae* infection.

## CASE REPORT

A 25-year-old African-American male with a history of diabetes presented to our emergency department (ED) with eye irritation, painful mouth sores, and difficulty swallowing. Three weeks prior to this visit, he was seen at a local ED for a small abscess on the posterior neck and treated with a 10-day course of trimethoprim/sulfamethoxazole (TMP-SMX). He did not begin this antibiotic for three days and took it intermittently over the next three weeks. He returned to the local ED two weeks after the first visit for symptoms of sore throat and lip swelling. This was attributed to a food allergy, and he was discharged with a five-day course of prednisone. He returned to the same institution two days later with worsening lip swelling and new mouth sores. He was prescribed nystatin suspension for presumed oral candidiasis that he took with the prednisone and remaining TMP-SMX for two days prior to arriving at our ED.

**Image 1:**
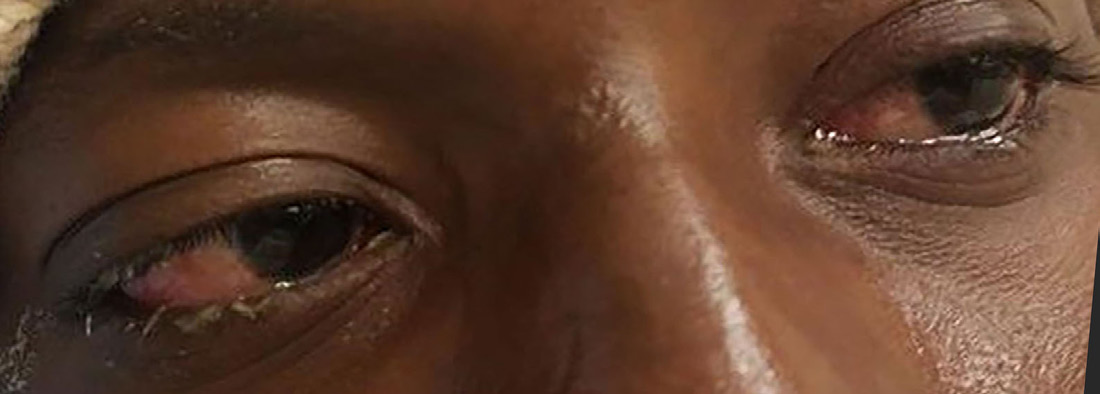
Injected conjunctiva with epithelial sloughing. Patient consent was given to use this photo.

**Image 2:**
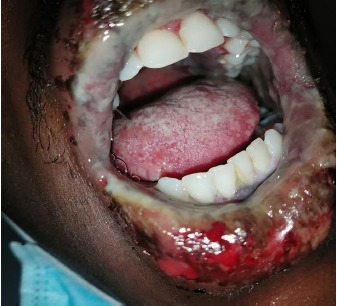
Severe stomatitis with diffuse ulcerations.

He arrived at our institution with two remaining tablets of TMP-SMX and complaining of worsening lip swelling, mouth sores, eye irritation, and difficulty swallowing over the two days since his last visit to the local ED. Vital signs on presentation were a temperature of 38.6 degrees Celsius, blood pressure 132/73 millimeters of mercury, heart rate 115 beats per minute, respiratory rate 16 breaths per minute, and pulse oximetry 99% on room air. His review of systems was positive for odynophagia, sore throat, and eye irritation; negative for cough, rash, joint pain, or genital irritation. Physical examination showed injected conjunctiva bilaterally with sloughing (Image 1); visual acuity was intact. The mouth and pharynx had severe stomatitis with ulcers involving the lips, tongue, buccal mucosa, and oropharyngeal mucosa (Image 2). There were no skin rashes and the lesion on his neck was well healed. All images were taken and published with the express, written consent of the patient.

Laboratory investigation revealed negative gonococcus/chlamydia polymerase chain reaction and negative* M. pneumoniae* immunoglobulin M on immunofluorescence assay. He was treated with intravenous (IV) fluids and admitted to the hospital for ophthalmologic and dermatologic consultations. The previously prescribed TMP-SMX was discontinued. He received IV methylprednisolone and mixed medication mouthwash for his stomatitis. The mild conjunctival sloughing was treated with erythromycin ophthalmic ointment, prednisolone acetate ophthalmic drops, and artificial tears, but did not require amniotic membrane graft. He was discharged after two days on a prednisone taper, prednisolone ophthalmic drops, and erythromycin ophthalmic ointment.

## DISCUSSION

SJS is part of a spectrum of mucocutaneous diseases affecting the skin and mucous membranes, which include erythema multiforme minor, SJS (erythema multiforme major), and toxic epidermal necrolysis.^5^ Skin involvement occurs in more than 90% of patients, but the appearance of skin lesions can vary from targetoid to diffuse erythema.^6^ Mucosal lesions typically occur at multiple sites such as the mouth, eyes, or genitalia. In adults, medications such as aromatic antiepileptics, allopurinol, sulfonamides, and nonsteroidal anti-inflammatory drugs are the most common precipitants.^5^,^6^ Bacterial and viral infections are the most commonly identified cause in children, with *M. pneumoniae* being the most common infectious agent associated with SJS among all age groups.^5^

**CPC-EM Capsule**

What do we already know about this clinical entity?

*Rare cases of incomplete Stevens-Johnson syndrome having only mucositis without rash occur in children infected with* Mycoplasma pneumoniae.

What makes this presentation of disease reportable?

*We report the first case of oral and ocular SJS without skin lesions in a healthy adult after exposure to an antibacterial sulfonamide and without* Mycoplasma pneumoniae* infection.*

What is the major learning point?

Pay careful attention to new or recently completed medications in patients complaining of skin or isolated mucous membrane complaints.

How might this improve emergency medicine practice?

Awareness that incomplete Stevens-Johnson syndrome can occur in adults as well as children will help clinicians identify precipitating agents and avoid delays in diagnosis.

Mucositis without skin involvement is an extremely rare variation of SJS most often seen in children infected by *M. pneumoniae.*^4^,^7^ Isolated mucositis is also a rare complication associated with combining TMP-SMX and methotrexate.^8^ However, our case is the first reported instance of isolated mucositis in an adult, brought on by a single medication known to cause SJS without clinical or laboratory evidence of *M. pneumoniae* infection. While some authors feel these atypical or incomplete presentations of SJS represent a distinct clinical entity, a common immunologic mechanism involving interleuken-15 and cluster of differentiation 8+ cytotoxic T cell-induced apoptosis of keratinocytes is likely common to all forms of SJS.^9^

Multiple outpatient visits were required in this case to establish the correct diagnosis. Additionally, we were assisted by obvious vital sign abnormalities and symptoms that precluded discharge. Careful attention to new or recently completed medications will often provide diagnostic clues to help make such a difficult diagnosis. In this case, the diagnosis was made when the provider found the bottle of TMP-SMX in the patient’s medication bag.

Treatment of SJS is largely supportive and includes removal of any offending medications. Corticosteroids, cyclosporine, IV immunoglobulin, plasmapheresis, and tumor necrosis factor inhibitors have all been used.^10^, ^11^ There is no clear benefit to any pharmacologic strategy, and treatments should be individualized based on severity and timing of the symptoms. Patients with severe conjunctival sloughing should receive amniotic membrane grafting early in their disease to preserve visual acuity.^12^ Our patient improved with removal of the offending antibiotic and steroids. He was advised to avoid other antimicrobial sulfonamides.
